# Beyond Fungitoxicity: Recent Achievements in Targeted Fungicide Discovery

**DOI:** 10.3390/jof12060446

**Published:** 2026-06-18

**Authors:** Xiyu Wu, Jianping Lu, Shoucai Ma, Fucheng Lin, Xuetao Shi

**Affiliations:** 1 Xianghu Laboratory, Hangzhou 311231, China; wuxiyu@xhlab.ac.cn (X.W.); jplu@zju.edu.cn (J.L.); mashoucai@xhlab.ac.cn (S.M.); 2 College of Life Sciences, Zhejiang University, Hangzhou 310058, China; 3 State Key Laboratory for Quality and Safety of Agro-Products, Zhejiang Provincial Key Laboratory of Agricultural Microbiomics, Key Laboratory of Agricultural Microbiome (MARA), Institute of Plant Protection and Microbiology, Zhejiang Academy of Agricultural Sciences, Hangzhou 310021, China

**Keywords:** phytopathogenic fungi, fungicide resistance, targeted fungicide discovery, anti-virulence, host defence, structure-based design

## Abstract

Phytopathogenic fungi pose a constant threat to worldwide agricultural production. Given the widespread development of fungicide resistance and increasing environmental and regulatory constraints, precision disease-control strategies are urgently needed to enhance selectivity, durability, and sustainability. This review critically examines recent advances in targeted fungicide discovery against phytopathogenic fungi. We categorize these strategies into three complementary groups: (1) targeting fungal biological processes that are essential or infection-associated; (2) disarming pathogen virulence by interfering with immune evasion and effector-mediated interactions; and (3) activating or redirecting host defence through host-directed or dual-action interventions. We compare these strategies with respect to mechanistic rationale, expected selectivity, resistance risk, and field-deployment challenges. Additionally, we discuss emerging enabling technologies—including compound repurposing, structural biology, and artificial intelligence-assisted fungicide design—that accelerate target identification and lead optimization. These strategies have begun to facilitate the discovery of compounds with improved specificity and disease-control potential. We believe that the integrated application of these approaches may support the development of more selective and potentially durable disease-control agents.

## 1. Introduction

Fungal pathogens remain a major threat to crop production and food security. They cause substantial yield losses in many important crops, and fungicides therefore remain essential for disease management, yield protection, and postharvest quality preservation [1]. However, the long-term effectiveness of conventional fungicides is under increasing pressure. Resistance has become widespread in many mode-of-action groups, particularly when disease control relies on a limited number of molecular targets. At the same time, environmental concerns and regulatory restrictions are placing further constraints on the use of broad-spectrum active ingredients [2].

These pressures have increased interest in more selective strategies for fungal disease control. Such strategies can be broadly grouped into three directions. The first is to target fungal biological processes that are essential for growth, infection, or stress adaptation, particularly when these processes provide opportunities for selective chemical intervention. The second is to disrupt pathogen virulence factors, thereby reducing the ability of the pathogen to evade host defence or cause disease. The third is to enhance host immunity, with the aim of improving the plant’s capacity to recognize, restrict, or tolerate infection. Although these strategies differ in their immediate targets, they share a common goal: to move beyond disease control based mainly on broad-spectrum fungitoxicity and toward more precise, mechanism-based interventions.

In this review, we discuss representative advances in these three areas, including target discovery and selected examples of lead compounds or intervention strategies. We also highlight enabling approaches that are increasingly used in targeted fungicide research, such as compound repurposing, structure-based design, and artificial intelligence-assisted discovery. This review does not aim to provide an exhaustive catalogue of all possible targets or technologies. Instead, it summarizes several active directions in targeted fungicide discovery and discusses their potential and current limitations.

Here, we use “targeted fungicide discovery” in a specific and operational sense. It does not simply mean that a fungicide has a known molecular mode of action, because many conventional fungicides already act on defined targets such as sterol biosynthesis, mitochondrial respiration, tubulin polymerization, or cell-wall biosynthesis. Rather, we use the term to describe mechanism-guided discovery strategies that deliberately exploit fungal biology, pathogen virulence mechanisms, or host–pathogen interactions or host defence to improve disease-control precision, selectivity, durability, or deployment flexibility.

Under this definition, “targeted” may refer to several related but distinct dimensions: improved taxonomic or pathosystem selectivity; reduced non-target or host toxicity; inhibition of infection-associated processes rather than general fungal growth alone; disarming of pathogen virulence or immune evasion; host-directed or dual-action disease protection; and rational discovery workflows enabled by structural biology, compound repurposing, or AI-assisted design. Established fungicide targets are discussed in this review only when recent studies revisit them through new chemical scaffolds, distinct binding modes, structure-guided optimization, resistance-breaking potential, or improved selectivity.

Consistent with this definition, the primary focus of this review is targeted fungicide discovery for phytopathogenic fungi. Accordingly, most examples discussed below are drawn from fungal crop pathosystems or from studies directly addressing fungal targets, fungal virulence, or fungal disease control. In a limited number of cases, we also refer to studies from oomycetes, non-crop fungal systems such as yeast or human pathogenic fungi, medical mycology, or bacterial plant disease systems. These examples are not presented as direct evidence for fungicide development against phytopathogenic fungi, but are included only when they illustrate transferable concepts, such as structure-guided selectivity design, targeting extracellular virulence factors, or host-directed defence modulation.

## 2. Discovery of Fungicide Targets: From Broad-Spectrum Toxicity to Targeted Disease Control

This section does not aim to provide a complete list of all possible fungicide targets. Instead, it highlights representative target spaces that illustrate different routes toward more selective fungal disease control. The targets and pathways discussed below are therefore used as examples, rather than as a comprehensive catalogue.

### 2.1. Revisiting Established Fungicide Targets

The inclusion of established fungicide targets in this section does not imply that all conventional fungicides with known modes of action are considered “targeted” in the sense used in this review. Instead, these targets are discussed because recent studies have revisited them using target-informed approaches, including new scaffolds, altered binding modes, allosteric inhibition, structure-guided optimization, or strategies intended to overcome existing resistance. Thus, established targets are used here to illustrate how classical mode-of-action spaces can be re-examined within a more selective and mechanism-guided discovery framework.

Established fungicide targets—such as sterol biosynthesis (e.g., demethylation inhibitors, DMIs) [3], mitochondrial respiration (e.g., Quinone outside Inhibitors, QoIs; and Succinate Dehydrogenase Inhibitors, SDHIs) [4,5], and tubulin polymerization (e.g., benzimidazoles) [6]—have been foundational to plant disease control but are now compromised by severe, widespread resistance [7].

However, these established targets are not obsolete. Their value can be renewed by identifying inhibitors with new chemical scaffolds or distinct binding modes, such as allosteric binding, that may help overcome existing resistance [8]. Enprocymid (E8) provides a representative example. Li et al. [9] determined the cryo-EM structure of yeast succinate dehydrogenase (SDH), which supported the structure-based design of enprocymid as a potent and selective fungicide candidate against multiple phytopathogenic fungi, with reported high efficacy and reduced environmental toxicity. Similarly, deep learning was used to identify celestolide as a natural allosteric inhibitor of the *Botrytis cinerea* sterol 14α-demethylase CYP51 [8]. By disrupting ergosterol biosynthesis and membrane integrity, celestolide effectively protected strawberries against gray mold [8].

The fungal cell wall is essential for maintaining cell morphology, osmotic stability, and environmental interactions. It is primarily composed of chitin, β-glucans, and glycoproteins [10]; its components are typically absent in plants and mammals, making them attractive targets for highly specific fungicides. Chitin, a polysaccharide of β-1,4-linked N-acetylglucosamine (GlcNAc), is synthesized by chitin synthase (CHS) and is critical for fungal cellular integrity, making its biosynthesis pathway a promising drug target [11]. 

#### Methodological Analogies from Oomycete and Yeast Chitin Synthase Structures

Although *Phytophthora sojae* is an oomycete rather than a true fungus, structural studies of its chitin synthase PsChs1 have provided a useful comparative model for understanding directional chitin synthesis and inhibitor binding. Cryo-EM analyses of PsChs1 in multiple catalytic states, including apo, substrate-bound, and Nikkomycin Z(NikZ)-bound forms, offer a methodological analogy for structure-guided inhibitor design in fungal and oomycete pathogens [12]. Similarly, another research team revealed the directional, multistep mechanism of chitin synthesis by determining the high-resolution cryo-EM structure of *Saccharomyces cerevisiae* Chs1 in seven distinct states. These structures captured the enzyme in its apo form, as well as bound to substrates (UDP-GlcNAc; UDP-GlcNAc + GlcNAc), products (UDP; UDP + GlcNAc), and key peptidyl nucleoside inhibitors (Polyoxin B and NikZ) [13]. Complementing these structural advances, recent work highlighted the molecular basis for inhibition by NikZ [14], the antifungal agent long recognized for its bioactivity against plant pathogens [15].

β-(1,3)-glucan is another major component of the fungal cell wall, and its synthase, FKS1, is an important antifungal target [16]. Recent structural studies of FKS1 have provided key insights into its catalytic mechanism, regulation, and resistance-associated changes [16,17]. In addition to inhibiting glucan synthase, direct targeting of β-1,3-glucan itself may also be possible. For example, the plant-derived compound poacic acid was reported to bind β-1,3-glucan and showed activity against the fungal pathogen *Sclerotinia sclerotiorum* and *Alternaria solani* [18].

Taken together, these studies show that established fungicide targets can still provide valuable opportunities for discovery. However, their further development should be supported by improved structural information, stronger evidence of selectivity, or clearly distinct binding mechanisms. At the same time, target-level innovation alone is not sufficient. Resistance management, field performance, formulation suitability, and crop safety must also be evaluated carefully. Therefore, revisiting an established molecular target qualifies as targeted discovery in this review only when it is accompanied by a clear strategy for improving selectivity, overcoming resistance, modifying binding behaviour, or linking target engagement to disease-control precision.

### 2.2. Exploring the Targeting Potential of Pathogenicity-Related Pathways

From a biological perspective, many pathogenicity-related pathways provide attractive target spaces because they control processes required for infection, growth, stress adaptation, or host colonization. As summarized in Table 1, representative examples include the cell wall integrity pathway, MAPK pathways, cAMP–PKA signaling, autophagy, TOR signaling, the calcineurin pathway, and G protein signaling. However, biological importance alone is not sufficient to define a practical fungicide target. A useful target must also provide a realistic basis for selective chemical intervention.

A major challenge is that many pathogenicity-related pathways are highly conserved across eukaryotes. This conservation raises important safety concerns. If selectivity is insufficient, inhibition of these pathways may affect the plant host or other non-target organisms. Therefore, promising targets may include not only pathways that are unique to fungi, but also conserved pathways in which fungal proteins show exploitable structural differences from their plant or animal homologues. In this context, targets such as Mps1 [19] and calcineurin [20] remain worthy of attention, provided that their selectivity is rigorously validated.

#### Conceptual Analogy from Human Pathogenic Fungi: Calcineurin-Selective FK520 Analogs

Studies on calcineurin-selective FK520 analogs in human pathogenic fungi provide a methodological analogy rather than direct crop-pathogen evidence. These studies illustrate how structure-guided design can help separate antifungal activity from potential host toxicity, a principle that may be relevant when evaluating conserved targets such as calcineurin in phytopathogenic fungi [21]. More broadly, these findings suggest that useful targets should not be limited to pathways that are completely absent from the host. Conserved but essential biological processes may also be considered if sufficient structural divergence can be demonstrated and if the target is chemically tractable. Thus, the central question is not simply whether a pathway is required for pathogenicity, but whether it can be targeted with an acceptable balance between efficacy and selectivity.

**Table 1:** Representative pathogenicity-related pathways as potential targets for targeted fungicide discovery.

Targets/Pathway	Description	Targetability *	Refs.
Cell Wall Integrity (CWI) pathway	The CWI pathway, the primary regulator of cell-wall biosynthesis in fungi, is essential for the pathogenicity of many phytopathogens	Moderate to high. High-resolution cryo-EM and crystal structures have been resolved for the pathway’s core synthases (CHS, FKS1) as well as its regulatory kinases.	[12,16,22]
Mitogen-Activated Protein Kinase (MAPK) Pathways	MAPK signaling pathways regulate key life activities, including cellular responses to external stimuli, morphogenesis, cell wall integrity, and pathogenesis	Moderate to high. Multiple MAPK subtypes (e.g., PMK1, Mps1) have been genetically validated as essential for the pathogenicity of plant-pathogenic fungi; The crystal structure of MoMps1 has been resolved; although target validity is well established, selectivity and chemical tractability require further validation.	[23,24,25,26,27,28]
Cyclic Adenosine Monophosphate-Protein Kinase A (cAMP-PKA) Signaling Pathway	The cAMP-PKA pathway regulates multiple processes essential for pathogenicity, such as spore germination, hyphal growth, appressorium formation, toxin production, and environmental stress responses	Moderate. Genetic validation confirms that this pathway serves as a central regulatory node for fungal spore germination, appressorium formation, toxin biosynthesis, and infection and colonization; while the target demonstrates high validity, its targetability is constrained by its high conservation across eukaryotes.	[29]
Autophagy	Autophagy is crucial for eukaryotic survival under stress and regulates pathogenicity in various plant pathogenic fungi	Context-dependent. Several autophagy- or mitophagy-associated targets have yielded chemically validated leads in plant-pathogenic fungi (FK506 and Pan-RAS-IN-1), but selectivity and pathway-level pleiotropy remain important concerns.	[30,31,32,33]
Target of rapamycin (TOR) signaling pathway	The TOR signaling pathway is a central regulator of key pathogenic processes in plant pathogenic fungi and is a potential target for plant disease control	Context-dependent. Genetic validation confirms that this pathway serves as a core regulatory node for nutrient sensing, hyphal growth, sexual reproduction, and pathogenicity in plant-pathogenic fungi, demonstrating strong target validity.	[34,35]
Calcineurin (CaN) Pathway	The core enzyme of CaN Pathway, Calcineurin, a Ca^2+^/calmodulin-dependent serine/threonine phosphatase, plays a central role in fungal stress response, growth, morphogenesis, and pathogenicity	Moderate. Calcineurin serves as a core regulatory phosphatase in fungi, governing stress responses, ion homeostasis, morphogenesis, and pathogenicity; high-resolution structural analyses of the interaction interfaces among its catalytic subunit, regulatory subunit, and immunophilins are available, and the framework for its rational design is well-established.	[36,37]
The G protein signaling pathway	The G protein signaling pathway is a master regulator that is essential for the pathogenicity of plant pathogenic fungi	Context-dependent. Some fungus-specific G protein-coupled receptor (GPCR) families, such as Pth11-like receptors in *Magnaporthe oryzae*, may offer relatively high target specificity, although ligandability, redundancy, and functional validation remain important constraints.	[38,39]

* High: direct structural or chemical evidence exists and selectivity is plausible; Moderate: strong genetic/pathogenicity validation exists, but chemical tractability or selectivity remains uncertain; Context-dependent: target relevance is strong, but conservation, redundancy, or host toxicity may constrain development.

### 2.3. Discovering Novel Targets and Corresponding Lead Compounds

Although established fungicide targets remain valuable, further progress in fungicide discovery depends on identifying additional targets that can broaden the range of intervention strategies. Many potential targets have been proposed in recent years, but they differ substantially in their level of validation and translational potential [7,40]. Some are supported mainly by genetic or mechanistic evidence, whereas others have already yielded compounds with measurable antifungal activity or disease-control efficacy. Therefore, biologically interesting targets should be distinguished from those that have begun to show chemical tractability and practical relevance.

Table 2 summarizes representative target–compound pairs reported in recent targeted fungicide research and organizes them by validation evidence and experimental scale. Because the listed examples differ in pathogen/crop system, target-engagement evidence, biological assay type, and field-level support, they should be interpreted as representative cases with different levels of experimental and translational maturity rather than as equally advanced fungicide candidates. The table is therefore intended as evidence for major directions in targeted fungicide discovery, including fungal biology-targeted intervention, anti-virulence strategies, and host-directed or dual-action protection.

## 3. Action Mechanisms of Targeted Fungicides

**Figure 1:**
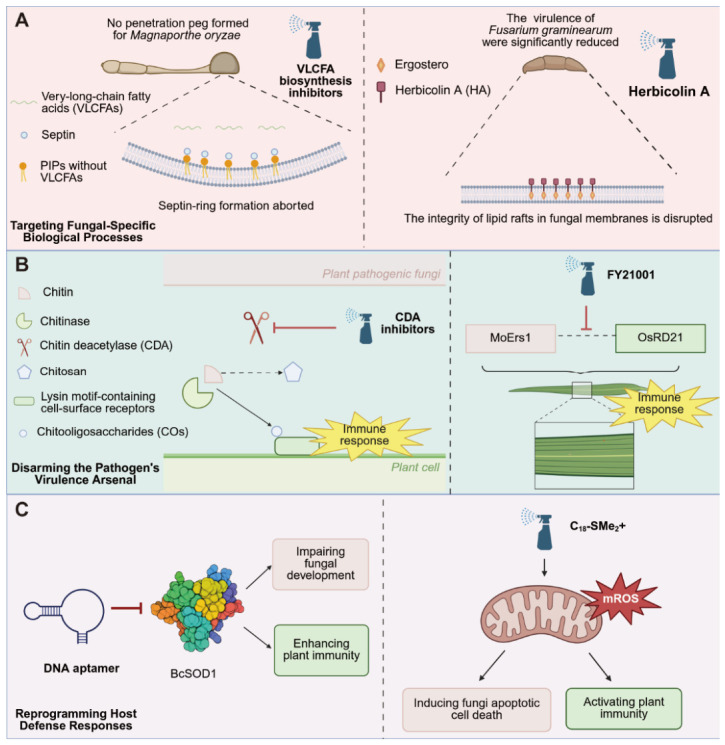
Three representative paradigms for the design and development of targeted fungicides. (**A**) Targeting fungal-specific biological processes. Targeting very-long-chain fatty acid (VLCFA) biosynthesis (Left panel): Inhibiting VLCFA biosynthesis disrupts pathogenesis in *M. oryzae*. VLCFAs inhibitors prevent the synthesis of VLCFA-containing phosphatidylinositol phosphates (PIPs), which are essential for septin ring assembly and penetration peg development; Disrupting pathogen integrity (Right panel): The compound herbicolin A (HA) binds ergosterol, disrupting ergosterol-containing lipid rafts in *F. graminearum*. This disruption suppresses hyphal growth, severely impairs perithecium formation, and attenuates virulence. (**B**) Disarming the pathogen’s virulence arsenal. Blocking immune evasion (Left panel): The fungal enzyme chitin deacetylase (CDA) converts chitin to chitosan to evade plant immune detection. Inhibiting CDA activity therefore restores host’s ability to detect the pathogen; Neutralizing effector proteins (Right panel): The *M. oryzae* effector MoErs1 suppresses host immunity by inhibiting the host papain-like cysteine protease OsRD21. The inhibitor FY21001 specifically disrupts the MoErs1-OsRD21 interaction, thereby restoring host defence. (**C**) Reprogramming host defence responses. Employing nucleic acid aptamers (Left panel): The DNA aptamers SOD9.14F and SOD9.26F target the BcSOD1 enzyme in *B. cinerea*, significantly inhibiting fungal germination and disease progression while simultaneously priming plant defence responses; Targeting mitochondrial function (Right panel): Mono-alkyl lipophilic cations (MALCs), particularly the novel compound C_18_-SMe_2_^+^, exhibit potent antifungal activity by disrupting mitochondrial function. This disruption induces mitochondrial reactive oxygen species (mROS) production, inhibits oxidative phosphorylation, and triggers fungal apoptosis, while concurrently priming plant defence responses.

Modern fungicide development is moving beyond direct fungitoxicity. It increasingly aims to target defined fungal biological processes, suppress infection by disarming pathogen virulence, or reprogram host defence to limit fungal colonization. These strategies have expanded the conceptual scope of targeted fungicide discovery, but they differ in their translational maturity and practical feasibility (Figure 1).

The three intervention modes—targeting fungal biology, disarming pathogen virulence, and enhancing host immunity—differ in their mechanistic basis, expected selectivity, resistance risk, and deployment challenges. Fungal biology-targeted strategies may offer broad-spectrum potential, but they can face selectivity concerns when the target is conserved. Anti-virulence strategies may reduce disease without strong direct fungitoxicity, but their efficacy can depend on host context and may be limited by functional redundancy. Host-directed strategies can enhance plant resistance, but their performance may vary with host genotype, treatment timing, and delivery efficiency. Comparing these modes helps clarify their complementary roles and limitations in targeted fungicide discovery.

### 3.1. Targeting Fungal-Specific Biological Processes

This strategy focuses on biological processes that are specific to fungi or that play critical roles during infection. Its main goal is to improve selectivity by targeting functions that directly support pathogen development or host invasion, rather than relying solely on broad-spectrum cytotoxicity. In many plant-pathogenic fungi, infection depends on specialized developmental events, including appressorium formation, host penetration, and membrane reorganization [60]. These processes provide useful entry points for targeted intervention.

For many appressorium-forming fungi, including *M*. *oryzae* and several *Colletotrichum* species, melanized appressoria contribute to host penetration by supporting infection-cell function, turgor generation, or appressorial cell-wall rigidity [61,62]. In *Colletotrichum* spp., melanin-associated appressorium function has been reported in systems such as *C. gloeosporioides* and *C. graminicola*, although the precise contribution of melanin can differ among species and pathosystems [62,63,64]. Consequently, melanin biosynthesis remains an important infection-related target space, but its target value should be interpreted in a pathogen-specific manner.

Conventional melanin biosynthesis inhibitors, such as tricyclazole and related compounds, interfere with enzymes in this pathway and impair appressorium function [65,66]. More recent work has shown that guaiacol can inhibit DHN-melanin biosynthesis in *Exserohilum turcicum* and reduce northern corn leaf blight in pot experiments [45]. These findings indicate that melanin biosynthesis remains a useful infection-related target. However, resistance to some older melanin biosynthesis inhibitors has been reported, showing that this strategy is also vulnerable to adaptation in field populations [67].

VLCFAs provide another example of an infection-associated target space. VLCFAs are involved in fungal cell-shape regulation [68] and are also linked to septin-dependent infection processes [51]. In *M. oryzae*, inhibitors of VLCFA biosynthesis, including metazachlor, cafenstrole, and diallate, disrupt septin ring formation and interfere with pathogenic development [51]. These compounds also showed control of rice blast under field conditions [51]. This example is important because it links a defined metabolic process to a specific infection-related structure, rather than to general growth inhibition alone. However, the broader applicability of this approach to other plant disease systems, as well as its selectivity under practical conditions, still requires careful evaluation.

Fungal membrane organization has also emerged as a potential point of intervention [69]. Herbicolin A (HA), produced by *Pantoea agglomerans* ZJU23, was reported to bind ergosterol and disrupt ergosterol-containing lipid rafts in fungal membranes [53]. In *Fusarium graminearum*, this disruption affects hyphal growth, perithecium formation, and pathogenicity [53].

Taken together, these examples show that fungal-specific or infection-associated biological processes provide valuable target space for fungicide discovery. Their main value lies in targeting processes that are closely linked to disease initiation and progression, rather than causing only general toxicity. However, their practical development remains uneven. Even when a target has a clear mechanistic rationale, broader challenges such as resistance management, formulation suitability, field stability, and crop safety still need to be addressed.

### 3.2. Disarming the Pathogen’s Virulence Arsenal

Anti-virulence strategies [70] aim to reduce disease by disrupting functions that are required for pathogenicity, rather than by directly inhibiting fungal growth [71]. In plant-pathogenic fungi, these functions include immune evasion, manipulation of host targets, and secretion of virulence factors. This strategy has attracted increasing attention because it may provide an alternative or complement to conventional fungicides, particularly under widespread fungicide resistance.

Chitin deacetylases (CDAs) are among the clearest examples of virulence-associated enzymes that can be considered for anti-virulence intervention [72]. Chitin normally acts as an immunogenic fungal cell wall component, but some pathogens convert it to chitosan through deacetylation, thereby reducing host recognition [73]. This role in immune evasion, together with the absence of CDAs from plants and mammals, has made them attractive targets for selective inhibition. Several types of CDA inhibitors have been reported, including benzohydroxamic acid [54], compounds identified through QSAR-guided design [55], and inhibitors obtained by structure-based virtual screening [56]. These studies show that CDA inhibition is chemically approachable and can reduce disease without necessarily acting as a conventional fungitoxic mechanism. However, the limitations of this strategy should also be stated clearly. Because CDA inhibition mainly exposes the pathogen to host defence rather than directly killing it, its effectiveness is likely to depend on the ability of the host to respond. In susceptible cultivars or under high disease pressure, this dependence may reduce practical efficacy. For this reason, CDA inhibitors may be more useful as part of a broader control strategy than as a universal standalone solution.

Fungal effector proteins are another important anti-virulence target class. These secreted proteins act directly at the host–pathogen interface and help suppress immunity or alter host physiology in ways that favour infection [74,75]. A representative example is the *M. oryzae* effector MoErs1, which interferes with the host immune protease OsRD21 [57]. Based on the structural interface between these proteins, FY21001 was developed to disrupt their interaction and to restore host defence, providing a useful proof of concept for effector-targeted intervention [57]. At the same time, strategies targeting effectors face significant biological constraints. Plant pathogenic fungi often deploy a diverse array of effectors [74,75]; consequently, inhibiting merely a single one may yield significantly diminished results due to functional redundancy. Furthermore, effector genes are frequently situated within rapidly evolving regions of the genome, a factor that could curtail the effective lifespan of highly specific inhibitors. While these considerations do not entirely invalidate effector-targeting strategies, they suggest that simultaneously inhibiting multiple functionally similar effectors may represent a viable approach.

A common assumption is that anti-virulence strategies may impose less selection pressure than conventional fungicides because they are often not strongly fungitoxic in vitro. This point should be treated cautiously in the context of crop protection. If an anti-virulence compound reduces infection and prevents pathogen reproduction in the host, selection is still being applied, even if the mechanism differs from direct growth inhibition. Resistance may therefore still emerge, for example through redundancy, altered target interfaces, or compensatory changes in virulence programmes. Anti-virulence strategies should therefore be viewed as a different resistance problem, not as the absence of one.

Taken together, anti-virulence strategies offer useful new perspectives for targeted fungicide discovery. Their main value lies in shifting intervention from general fungal survival to pathogenicity-associated functions. However, their performance is likely to depend on the host background, pathogen biology, and the specific function being targeted. Careful evaluation of selectivity, durability, and application context is therefore essential before these strategies can be translated into practical disease-control tools.

### 3.3. Reprogramming Host Defence Responses

Reprogramming host defence represents a host-directed strategy for fungal disease control. In this framework, the immediate target is the plant rather than the pathogen alone. Disease protection may be achieved by strengthening immune perception, priming defence signalling, or combining direct pathogen inhibition with host activation. Compared with conventional fungicides, this strategy places greater emphasis on the contribution of the host to disease suppression. Its practical application, however, depends on whether the induced defence responses are sufficiently strong, timely, specific, and physiologically tolerable.

DNA aptamers provide one example of a host-directed or dual-action strategy. The aptamers SOD9.14F and SOD9.26F were reported to target *B. cinerea* superoxide dismutase 1 (BcSOD1). They inhibit fungal germination and disease progression while also activating defence-related responses in the plant [58]. This example is notable because it combines direct interference with a pathogen-associated target and stimulation of host defence within a single intervention. Despite this promise, aptamer-based crop protection still faces practical barriers. In particular, nucleic acid aptamers may be susceptible to nuclease-mediated degradation, and their stability, delivery efficiency, persistence on plant surfaces, and formulation requirements must be evaluated under field-relevant conditions [76,77]. Therefore, aptamer-based candidates should be assessed not only for target binding and disease suppression, but also for environmental stability and realistic delivery to infection sites.

A second example is provided by mono-alkyl lipophilic cations such as C_18_-SMe^2+^ [59]. These compounds were reported to disrupt mitochondrial function in several fungal pathogens, inhibit oxidative phosphorylation, and induce apoptosis-like responses, while also promoting defence-associated oxidative responses in the host [59]. This type of dual activity is of interest because it does not rely solely on either direct fungitoxicity or host stimulation. However, this dual mode of action also introduces complexity into the assessment process, as selectivity is often difficult to clearly define within complex biological systems.

Host-derived protein elicitors represent another possible route for enhancing resistance [78]. In rice, the secreted protein OsSSP1 was shown to activate immune signalling through the OsSSR1–OsBAK1 receptor complex, and its application conferred resistance to *M. oryzae* for a limited period without an obvious yield penalty [78]. This instance demonstrates that, theoretically, endogenous plant proteins can be utilized to bolster plant defence responses. However, the current evidence base supporting such strategies remains tenuous, and the generalizability of these research findings remains unclear.

Taken together, host-directed strategies can be implemented through several modalities, including nucleic acids, small molecules, and proteins. Their main advantage is that they may reduce exclusive reliance on direct pathogen toxicity and, in some cases, provide a broader basis for disease suppression. However, their limitations are equally clear. Efficacy may depend strongly on host genotype, developmental stage, treatment timing, and environmental conditions. Practical application may also be limited by delivery efficiency, environmental stability, production cost, and potential unintended effects on plant physiology. For this reason, host-immunity-based strategies should be assessed not only for biological activity, but also for robustness, crop safety, and feasibility under agricultural conditions.

### 3.4. Conceptual Analogies from Non-Crop-Pathogen Systems

#### 3.4.1. Conceptual Analogy from Medical Mycology: Membrane-Targeting Antifungal Compounds

Studies in medical mycology, including work on mandimycin, further suggest that fungal membrane phospholipids can be chemically tractable antifungal targets [79]. Although these studies are not derived from crop pathosystems, they provide a methodological analogy for exploring membrane-associated selectivity in phytopathogenic fungi. These findings suggest that fungal membrane organization may offer additional opportunities for targeted antifungal or fungicide discovery. However, as with other membrane-targeting strategies, important questions remain regarding selectivity, delivery efficiency, and performance under agricultural conditions.

#### 3.4.2. Conceptual Analogy from Oomycete Pathosystems: Targeting Extracellular Virulence Factors

Although outside fungal pathosystems, oomycete Nep1-like proteins (NLPs) provide a useful conceptual analogy for anti-virulence intervention [80]. Small molecules such as 6G7 have been reported to bind NLPs, block NLP-induced necrosis, and reduce disease-related effects in Phytophthora infestans [80]. Because oomycetes are phylogenetically distinct from fungi, this example should not be interpreted as direct evidence for targeted fungicide development against phytopathogenic fungi [80]. Instead, it illustrates the broader principle that extracellular virulence factors may be chemically targeted to reduce disease.

#### 3.4.3. Conceptual Analogy from Bacterial Plant Disease Systems: Host-Directed Defence Modulation

As a non-fungal conceptual analogy, peptide-based strategies provide an illustrative example of host-directed defence modulation. In citrus Huanglongbing (HLB), an antiproteolysis peptide was designed to stabilize the defence regulator MYC2 by interfering with its degradation, thereby enhancing jasmonate signalling and improving disease resistance [81]. Although this work was carried out in a bacterial pathosystem rather than a fungal one, it is discussed here only as a transferable host-directed design principle rather than as direct evidence for fungal disease control.

## 4. Accelerating Discovery: Innovative Approaches to the Discovery and Design of Targeted Fungicides

Recent advances in drug repurposing, structural biology, and artificial intelligence are reshaping targeted fungicide discovery and design. These approaches support different stages of the discovery pipeline. Drug repurposing accelerates early lead identification, structural biology clarifies target mechanisms and binding modes, and artificial intelligence-assisted methods improve structure prediction, virtual screening, compound prioritization, and candidate design. However, these approaches should be viewed as enabling tools. Their outputs still require biochemical validation, biological testing, and evaluation under agricultural conditions.

### 4.1. Repurposing Drugs

Human therapeutic compounds have attracted interest as starting points for fungicide discovery because they often come with existing pharmacological and chemical information. Propranolol is one representative example. It was identified as an inhibitor of phosphatidate phosphatase Pah1 and was shown to disrupt fungal lipid metabolism [50]. Subsequent chemical modification generated a derivative with improved activity and reported field efficacy against rice blast and *Fusarium* head blight [50]. Doxorubicin is another example. It was reported to inhibit phosphatidylserine decarboxylase and impair fungal development and virulence by disrupting phospholipid metabolism [82]. These cases indicate that compounds originally developed for human medicine can provide useful entry points for antifungal research. However, prior medical use does not replace the need for target-selectivity assessment, crop-safety evaluation, and validation under agricultural conditions.

Agrochemicals developed for other purposes can also be repurposed for fungicide discovery. Inhibitors of VLCFA biosynthesis, including cafenstrole, metazachlor, and diallate, were originally associated with herbicide-related chemistry and were later shown to interfere with septin-dependent infection processes in *M. oryzae* [51]. This case shows that existing agrochemical scaffolds can be redirected toward fungal infection-related processes. Natural products and endogenous regulators provide another source of leads. Melatonin and its derivatives were reported to target conserved fungal proteins, including Mps1, MoIcl1, and Cap20, and to suppress fungal pathogenicity [42,43,44]. These examples suggest that repurposing can support both lead identification and target exploration by linking existing chemistries to newly recognized infection-related mechanisms.

Taken together, these examples indicate that repurposing can accelerate early fungicide discovery. Its main value lies in using compounds with existing chemical or biological information, thereby reducing the need to begin from unexplored chemical space. Nevertheless, repurposed compounds must still be evaluated for target selectivity, differential sensitivity among pathogens, crop compatibility, and performance under agricultural conditions. Thus, repurposing should be viewed as a strategy for lead identification and target exploration, not as evidence that a compound is already suitable for field deployment.

### 4.2. Structural Biology-Driven Fungicide Discovery and Design

Structure-based design, often referred to as structure-based drug design (SBDD), has become an increasingly useful approach in fungicide discovery, particularly in target-focused stages such as hit identification and hit-to-lead optimization [83]. Although SBDD is more established in human drug discovery, it is increasingly being applied in agrochemical research [28,55,83,84,85]. Its main value is to define binding sites, clarify inhibition mechanisms, and guide the design of compounds with improved activity and selectivity.

Several recent studies illustrate how structural information can support fungicide discovery. One example is FY21001, which was developed on the basis of the crystal structure of the *M. oryzae* effector MoErs1 and a docking model of the MoErs1–OsRD21 interaction [57]. Similarly, researchers leveraged the resolved structure of Tps1 [84,86], a central metabolic integrator in *M. oryzae* [87]. Through virtual screening, they identified A1-4, a novel dual-specificity inhibitor that simultaneously targets trehalose-6-phosphate synthase (TPS) and trehalose-6-phosphate phosphatase (TPP). This unique multi-enzyme inhibition mechanism confers broad-spectrum antifungal activity with minimal phytotoxicity [52]. These cases indicate that structural information can help support target-focused design, particularly when the target is not easily addressed by conventional phenotypic screening alone.

**Figure 2:**
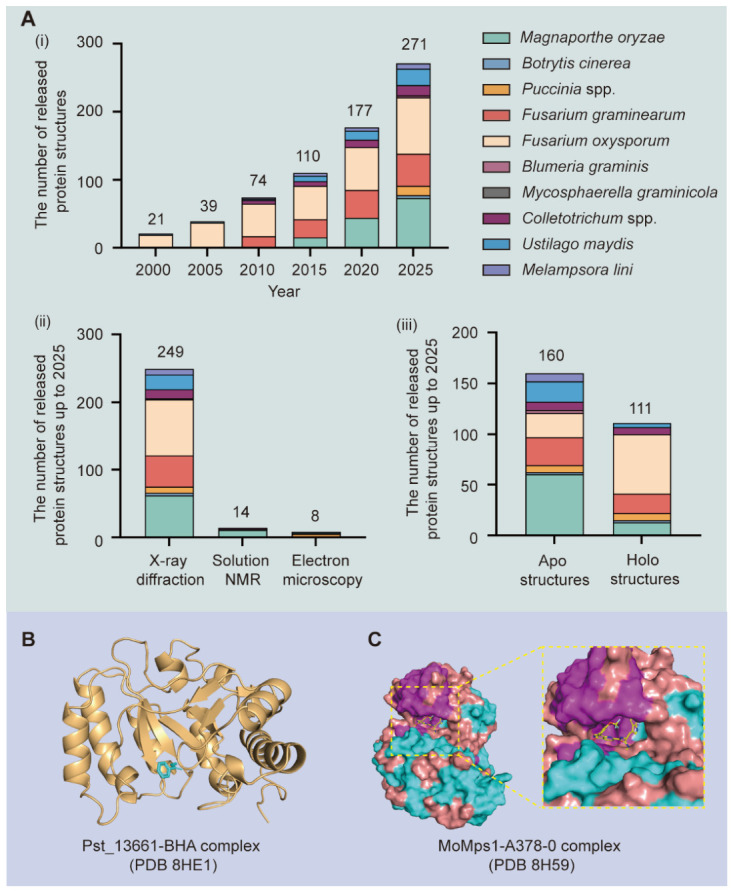
Protein structural landscape of the Top 10 fungal plant pathogens. (**A**) Bar graphs showing the summary of protein structures for the Top 10 fungal plant pathogens [88]. (i) The accumulation of protein structures deposited up to 2025; PDB retrieval date: 1 January 2026; Species retrieval keywords: *Magnaporthe oryzae*, *Botrytis cinerea*, *Puccinia* spp., *Fusarium graminearum*, *Fusarium oxysporum*, *Blumeria graminis*, *Mycosphaerella graminicola*, *Colletotrichum* spp., *Ustilago maydis*, and *Melampsora lini*; All collected data were experimentally determined structures; (ii) The distribution of experimental methods used to determine their protein structures. A total of 271 protein structures were analyzed (up to 2025); (iii) The proportion of protein structures in apo (unbound states) versus holo (ligand-bound states). A total of 271 protein structures were analyzed (up to 2025). (**B**) Experimental structure of the benzohydroxamic acid (BHA)–chitin deacetylase Pst_13661 complex (PDB 8HE1). (**C**) Experimental structure of the compound A378-0 in complex with MoMps1 (PDB 8H59). The compound-binding pocket is highlighted by a yellow dashed box in the enlarged view.

At the same time, the usefulness of SBDD depends on the availability of reliable three-dimensional structural information. In the Protein Data Bank (PDB), experimentally determined structures from major fungal plant pathogens have increased steadily over the past two decades. As summarized in Figure 2, a survey of reported structures (Data as of 1 January 2026) for the Top 10 fungal plant pathogens [88] identified 271 protein structures up to 2025, of which most were determined by X-ray diffraction, whereas only a small fraction were obtained by electron microscopy. Among these structures, 111 were reported in ligand-bound form. However, only a limited number represent complexes with fungicides or inhibitor molecules. Examples include the Pst_13661–BHA complex [54] and the MoMps1–A378-0 complex [28].

Taken together, these studies show that structural biology is providing a more detailed basis for fungicide discovery, both by clarifying target mechanisms and by improving the interpretation of compound binding. However, structural availability alone does not ensure that a target will be easy to exploit chemically, nor does it guarantee field-relevant efficacy.

### 4.3. AI and Computational Biology in Fungicide Discovery

AI and related computational methods are increasingly used in fungicide discovery, particularly in target-focused stages such as structure prediction, virtual screening, and candidate design. However, these methods cannot replace the necessity of biochemical validation or evaluation under agricultural conditions.

**Figure 3:**
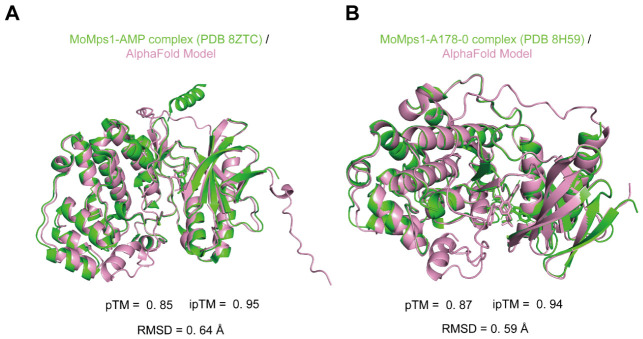
Applications of AI in targeted fungicide discovery. (**A**) Prediction of a ligand-bound complex: Structural superimposition of the experimental MoMps1-AMP complex (PDB 8ZTC) (green) with its AlphaFold3 predicted structure (pink), demonstrating high modeling accuracy (pTM = 0.85; RMSD = 0.64 Å). In this example, the AlphaFold3 model reproduced the AMP-binding site in the kinase pocket with high structural similarity to the experimental complex (ipTM = 0.95). (**B**) Prediction of an inhibitor-bound complex: Structural superimposition of the MoMps1-A378-0 complex (PDB 8H59) (green) with the corresponding AlphaFold3 predicted structure (pink) (pTM = 0.87; RMSD = 0.59 Å). The AlphaFold3 models reproduced the overall fold and ligand-binding pocket with high structural similarity to the experimental complexes (ipTM = 0.94).

One important application of AI in this field is protein structure prediction. AlphaFold2 [89] and AlphaFold3 [90] have made it easier to obtain structural models for targets that lack experimental structures, and these models can support subsequent screening or design work. In the case of MoMps1, comparison between predicted and experimental complexes suggested that AlphaFold3 could reproduce both overall structural features and ligand-binding pockets with useful accuracy (Figure 3). Such results indicate that AI-based prediction can provide practical support for target-focused discovery, particularly when experimental structural information is limited. At the same time, predicted structures should still be interpreted with caution.

AI is also being applied to large-scale virtual screening of chemical libraries [91,92]. Methods such as KarmaDock and DeepDock have been developed to improve the efficiency and accuracy of protein–ligand docking, and they may help reduce the number of compounds that need to be tested experimentally [93]. In this specific application scenario, the primary contribution of AI lies in optimizing the prioritization of compounds, rather than providing definitive evidence of their antifungal activity. Consequently, following the acquisition of screening results, subsequent experiments remain essential to conclusively validate the compounds’ target-binding capabilities, antifungal activity, and efficacy in controlling fungal diseases.

A further application of AI is de novo design. Tools such as RFdiffusion [94] and BindCraft [95] have expanded the range of approaches that can be used to generate candidate binders or peptides for defined targets. In the context of fungal disease control, these methods are of interest because they may eventually help address targets that are difficult to approach with conventional small-molecule chemistry, including protein–protein interactions. However, this field is currently still in its nascent stage and awaits extensive experimental validation.

Among current AI applications, structure prediction is already supporting target assessment and screening, while virtual screening mainly improves prioritization efficiency. De novo design of binders or peptides remains at an early stage, with limited experimental validation in crop protection. Therefore, the maturity and evidence level of these approaches differ, and their outputs should be interpreted accordingly.

Taken together, AI and computational biology are providing useful support for several stages of fungicide discovery, from structural modelling to compound prioritization and candidate design. Their main contribution is to improve efficiency and expand the range of testable ideas. Their limitations are equally clear: computational outputs remain provisional until they are supported by experimental and translational evidence. For this reason, AI is best regarded as an enabling approach within fungicide discovery rather than as an independent solution to the broader problems of efficacy, selectivity, and field deployment.

## 5. Discussion

The continued spread of fungicide resistance, together with increasing environmental and regulatory constraints, makes it necessary to explore alternatives to conventional broad-spectrum disease control. In this review, we have discussed three related directions in current fungicide research: targeting fungal biological processes with improved selectivity, disrupting pathogen virulence, and strengthening host defence. We have also summarized representative compounds and target–compound pairs reported in recent studies. Together, these advances indicate that fungicide discovery is moving beyond direct fungitoxicity toward a broader set of mechanism-guided intervention strategies.

### 5.1. Practical Evaluation Framework for Targeted Fungicide Candidates

To make the proposed evaluation framework more explicit, we summarize a stepwise assessment pipeline for targeted fungicide candidates in Table 3. This framework is intended to connect early mechanism-guided discovery with agricultural deployment. It begins with target and pathway relevance, followed by biochemical target validation, compound activity, selectivity, crop safety, formulation and delivery feasibility, environmental robustness, resistance risk, compatibility with existing disease-management programmes, and field-level validation. The framework is not meant to impose a rigid sequence for all discovery programmes, because different intervention modes may require different assays. Instead, it provides a practical checklist for distinguishing mechanistically interesting leads from candidates with realistic translational potential.

The relative importance of each stage will depend on the intervention mode. For fungal biology-targeted compounds, biochemical target engagement, pathogen selectivity, and resistance risk may be central. For anti-virulence compounds, plant immune competence, target redundancy, and durability of the virulence interface should receive particular attention. For host-directed or dual-action strategies, crop safety, defence-associated physiological cost, treatment timing, and delivery efficiency are especially important. Therefore, the framework should be applied flexibly while maintaining a clear distinction between mechanistic proof of concept and field-deployable disease-control candidates.

Beyond this staged evaluation framework, agricultural deployment requires more detailed consideration of application strategy, formulation feasibility, environmental robustness, production cost, regulatory feasibility, and integration into existing disease-management programmes.

### 5.2. Translational Considerations for Agricultural Deployment

Mechanistic proof of concept does not necessarily indicate agricultural deployability. Targeted fungicide candidates that perform well in biochemical, in vitro, or controlled plant assays must still be evaluated for dose–response behaviour, delivery to infection sites, persistence, and reproducible efficacy under realistic crop-production conditions. These issues are particularly important for infection stage, anti-virulence, and host-directed strategies, whose efficacy may depend strongly on application timing and pathogen developmental stage.

Environmental robustness should also be considered early. Rainfastness, UV stability, temperature stability, degradation rate, and persistence on plant surfaces can strongly influence field performance [96]. These factors may be especially limiting for peptides, proteins, aptamers, natural products, or other biomolecular interventions, which may require specific formulation or delivery systems to maintain activity.

For nucleic acid-based modalities, nuclease degradation represents a specific stability barrier that may reduce persistence after field application [97]. Aptamers are known to be susceptible to nuclease-mediated degradation, and chemical modification or protective formulation may therefore be required before such molecules can be considered realistic crop-protection candidates [97]. Peptide- or protein-based candidates may face related challenges associated with proteolysis, UV exposure, temperature fluctuation, and loss of activity on leaf surfaces.

The desired spectrum of control should be balanced against selectivity and non-target effects. Highly specific compounds may reduce off-target toxicity but may have narrower practical use, whereas broader-spectrum compounds require more extensive assessment of impacts on beneficial fungi, microbial communities, and crop physiology. Crop safety and phytotoxicity should therefore be evaluated through visible injury, growth, reproductive development, physiological performance, and yield-related observations where relevant.

Finally, candidate compounds should be assessed within existing disease-management programmes. Formulation feasibility, production cost, storage stability, compatibility with adjuvants or tank mixtures, rotation or mixture potential, cross-resistance risk, and regulatory feasibility will strongly influence whether a mechanistically attractive lead can become a practical disease-control tool. These issues may be particularly important for complex biomolecules, peptides, aptamers, or AI-designed binders, for which synthesis, purification, scale-up, and formulation may be more demanding than for conventional small-molecule fungicides. Tank-mix compatibility should also be evaluated experimentally, because incompatible mixtures can cause precipitation, phase separation, foaming, nozzle blockage, reduced efficacy, or crop injury. Therefore, candidate products should be assessed not only as individual active ingredients but also in formulation contexts that reflect realistic spray programmes. Together, these considerations help distinguish molecular proof-of-concept leads from candidates with realistic field-deployment and disease-management potential.

### 5.3. Resistance and Deployment Risks of Anti-Virulence and Host-Directed Strategies

Anti-virulence and host-directed strategies are sometimes described as potentially less resistance-prone than conventional fungicides, but this assumption should be treated cautiously in crop protection. If an intervention reduces infection, suppresses pathogen reproduction, or changes the relative fitness of pathogen genotypes in planta, selection pressure can still occur even when direct fungitoxicity is weak in vitro. Therefore, resistance risk should be evaluated according to the effect on pathogen transmission and reproduction in the host, rather than only according to growth inhibition in culture.

For anti-virulence compounds, resistance may arise through several routes. Pathogens may alter the target interface, reduce compound binding, deploy redundant virulence factors, or compensate through alternative infection programmes. Effector-targeting strategies may be particularly vulnerable when the targeted effector is variable, dispensable, or functionally redundant. Similarly, inhibitors of immune-evasion enzymes or extracellular virulence factors may lose effectiveness if pathogens use parallel mechanisms to avoid recognition or cause host damage. These risks do not invalidate anti-virulence strategies, but they indicate that target redundancy, conservation of the virulence function, and the fitness cost of escape variants should be considered during candidate evaluation.

Host-directed strategies pose a different but related set of deployment risks. Because these interventions rely partly or entirely on the plant response, their efficacy may vary with host genotype, developmental stage, physiological status, treatment timing, and environmental conditions. In addition, defence activation can impose physiological costs if it is too strong, prolonged, or poorly synchronized with infection. Such costs may appear as growth suppression, delayed development, altered resource allocation, phytotoxic symptoms, or yield penalties. Therefore, host-directed candidates should be evaluated not only for disease reduction, but also for defence-associated physiological costs and crop performance under relevant production conditions.

Resistance monitoring and deployment design should therefore be adapted to the intervention mode. For anti-virulence compounds, monitoring should include target-sequence variation, changes in virulence-factor expression, sensitivity shifts, and evidence of compensatory infection mechanisms. For host-directed or dual-action strategies, monitoring should include consistency of protection across cultivars and environments, crop safety, and possible trade-offs between defence activation and yield. In practice, both types of strategies may be most effective when deployed in mixtures, rotations, or integrated programmes with conventional fungicides, resistant cultivars, and biological control agents. Such deployment may reduce reliance on any single intervention mechanism and improve durability under field conditions.

### 5.4. Cross-Cutting Challenges and Future Integration

In addition to these deployment considerations, several cross-cutting scientific challenges remain. Selectivity is a central issue. Many biologically important targets, including components of MAPK [23,24,25,26,27,28] and TOR-related pathways [34,35], are highly conserved across eukaryotes. For such targets, practical value depends not only on pathogenicity relevance, but also on whether fungal proteins contain structural or functional features that can distinguish them from homologues in the plant host or other non-target organisms. Demonstrating such differences is essential for selective compound development.

Durability and crop compatibility also remain important across different intervention modes. Anti-virulence strategies require careful resistance-management planning because their efficacy may still impose selection pressure in planta, whereas host-directed strategies must balance disease suppression with possible physiological costs [74,75]. These considerations reinforce the need to evaluate target conservation, functional redundancy, crop safety, delivery feasibility, and performance under agriculturally relevant conditions.

Future plant disease management may benefit from integrating multiple intervention strategies. Combinations of conventional fungicides, anti-virulence compounds, and host defence enhancers may provide stronger and more durable control than any single component alone, provided that their interactions are well understood. Such combinations may also help reduce chemical inputs and delay efficacy loss. However, formulation compatibility, interaction effects, crop safety, resistance-management value, and field performance must be carefully assessed.

Structural biology and computational methods will continue to support targeted fungicide discovery. High-resolution structures and AI-assisted prediction, screening, and design can help expand target space, prioritize compounds, and guide optimization. However, these tools do not replace experimental validation. Computational predictions, structural models, and virtual screening outputs should be integrated with biochemical validation, biological assays, crop-safety assessment, and field-level evaluation.

## 6. Conclusions

Current research on selective fungal targets, anti-virulence strategies, and host-directed protection shows that several routes for targeted fungicide discovery are being explored in parallel. None of these approaches is sufficient on its own, and each still faces practical limitations. Nevertheless, together they provide a broader basis for developing disease-control strategies that are more selective, adaptable, and potentially more sustainable than approaches based mainly on broad fungitoxicity.

As summarized in the proposed evaluation framework (Table 3), future targeted fungicide candidates should be assessed across multiple layers, including target relevance to infection or disease development, biochemical target engagement, fungal or pathosystem selectivity, chemical tractability, delivery and formulation feasibility, environmental robustness, resistance-management potential, crop safety, non-target effects, compatibility with existing disease-management programmes, and reproducible efficacy under greenhouse and field conditions. Such a framework can help distinguish biologically interesting targets from candidates with realistic translational potential.

Targeted fungicide discovery should therefore be viewed not simply as the identification of new molecular targets, but as an integrated strategy linking fungal biology, host–pathogen interactions, chemical intervention, resistance management, and field applicability. Future progress will require rigorous validation across biochemical, biological, formulation, and agronomic scales, together with rational integration of pathogen-directed inhibition, virulence disarmament, and host-directed protection.

## Figures and Tables

**Table 2:** Representative target–compound pairs reported in recent targeted fungicide research, classified by validation evidence and experimental scale.

Compound/Intervention	Target/Pathway	Pathogen/Crop or Assay System	Evidence for Target Engagement	Biological Assay	Experimental Scale of Plant/Field Assays	Intervention Logic	Key Limitation or Maturity Note	Refs.
A378-0	*Magnaporthe oryzae* mitogen-activated protein kinase (MoMps1)	*Magnaporthe oryzae*/rice blast-related assay	Biochemical; structural	In vitro assay; plant assay	Controlled plant systems	Fungal biology-targeted	Strong target-level evidence, but broader field performance and selectivity require further evaluation	[28]
TAK-733	MoMps1 (common docking site)	*Magnaporthe oryzae*/rice blast-related assay	Biochemical	In vitro assay; plant assay	Controlled plant systems	Fungal biology-targeted	Target validity supported, but structural evidence is more limited than A378-0	[41]
Melatonin	MoMps1	*Magnaporthe oryzae*/rice blast-related assay; broad-spectrum activity reported	Biochemical	In vitro assay; plant assay	Detached tissues and potted plants	Fungal biology-targeted	Selectivity and field stability require further evaluation	[42]
Melatonin	*Magnaporthe oryzae* isocitrate lyase (MoIcl1)	*Magnaporthe oryzae*/rice blast-related assay	Biochemical	In vitro assay; plant assay	Detached tissues and potted plants	Fungal biology-targeted	Synergistic disease-control potential reported, but translational maturity remains limited	[43]
Mt-23	Lipid droplet coating protein Cap20	*Magnaporthe oryzae*, *Botrytis cinerea*, *Sclerotinia sclerotiorum*, *Bipolaris maydis*, *Rhizoctonia solani* and *Fusarium graminearum*/plant infection assay	Biochemical	In vitro assay; plant assay	Detached tissues	Fungal biology-targeted	Compared to melatonin, it exhibits better antifungal activity, but lacks support from evidence in field trials.	[44]
Guaiacol	Melanin biosynthesis	*Exserohilum turcicum*/northern corn leaf blight-related assay	Chemical/analytical evidence	In vitro assay; plant assay	Detached tissues	Fungal biology-targeted	Field-level durability and resistance risk require evaluation	[45]
Ebselen	Atg4-mediated Atg8 processing	*Magnaporthe oryzae*/rice blast-related assay; *Botrytis cinerea*/gray mold-related assay	Biochemical	In vitro assay; plant assay	Detached tissues and potted plants	Fungal biology-targeted	Autophagy-related target; selectivity and pleiotropic effects require caution	[46]
H_2_S-mediated S-sulfhydration	MoAtg18 S-sulfhydration	*Magnaporthe oryzae*/rice blast-related assay	Genetic; chemical; functional evidence	In vitro assay; plant assay	Detached tissues and potted plants	Fungal biology-targeted	Practical delivery and dose control may be challenging	[47]
SP-141	*Magnaporthe oryzae* Trs85–Ypt1 interaction	*Magnaporthe oryzae*/rice blast-related assay	Molecular biological evidence	In vitro assay; plant assay	Detached tissues and potted plants	Fungal biology-targeted	Protein-interaction target; target engagement and field performance need further evaluation	[48]
FK506	*Magnaporthe oryzae* FK506-binding protein 1B (MoFpr1)	*Magnaporthe oryzae*/rice blast-related assay	Biochemical; structural	In vitro assay; plant assay; field trial	Detached tissues, potted plants and disease-control under field conditions	Fungal biology-targeted	Strong multi-level evidence, but selectivity and deployment feasibility require careful assessment	[32]
Quinofumelin	FgDHODHII/*de novo* pyrimidine or uracil biosynthesis	*Fusarium graminearum*	Biochemical	In vitro assay	No whole-plant or field evidence	Fungal biology-targeted	Target mechanism reported, but lacking plant or field evidence	[49]
Propranolol and optimized derivatives	*Magnaporthe oryzae* phosphatidate phosphatase (MoPah1)	*Magnaporthe oryzae*/rice blast-related assay; *Fusarium graminearum*/wheat head blight-related assay	Biochemical	In vitro assay; plant assay; field trial	Detached tissues, potted plants and disease-control under field conditions	Fungal biology-targeted	Repurposed human therapeutic scaffold; crop safety and optimization remain important	[50]
Celestolide	CYP51/lanosterol 14α-demethylase	*Botrytis cinerea*/strawberry gray mold-related assay	Biochemical	In vitro assay; plant assay	Postharvest plant system	Fungal biology-targeted	Allosteric inhibitor concept; broader pathogen spectrum and field validation require evaluation	[8]
Very-long-chain fatty acid (VLCFA) biosynthesis inhibitors: metazachlor, cafenstrole, and diallate	VLCFA biosynthesis	*Magnaporthe oryzae*/rice blast-related assay	Genetic; chemical; functional evidence	In vitro assay; plant assay; field trial	Detached tissues, potted plants and disease-control under field conditions	Fungal biology-targeted	Existing agrochemical scaffold; selectivity and crop-context constraints should be evaluated	[51]
A1-4	*Magnaporthe oryzae* trehalose-6-phosphate synthase 1 (MoTps1) and *Magnaporthe oryzae* trehalose-6-phosphate phosphatase (Tps2)	*Magnaporthe oryzae*/rice blast-related assay; broad-spectrum activity reported	Biochemical	In vitro assay; plant assay	Detached tissues	Fungal biology-targeted	Dual-specificity mechanism; field validation and resistance risk remain to be established	[52]
Pan-RAS-IN-1	*Magnaporthe oryzae* cytochrome c oxidase subunit 6 (MoCox6)-mediated mitophagy/mitochondrial homeostasis	*Magnaporthe oryzae*/rice blast-related assay	Biochemical	In vitro assay; plant assay; field trial	Detached tissues, potted plants and disease-control under field conditions	Fungal biology-targeted	Multi-level evidence including field trial, but selectivity and deployment feasibility need assessment	[33]
Herbicolin A	Lipid raft integrity in fungal membranes	*Fusarium graminearum*/Fusarium head blight related-assay; broad-spectrum activity reported	Chemical; structural/biophysical; imaging evidence	In vitro assay; plant assay; field trial	Detached tissues and disease-control under field conditions	Fungal biology-targeted	Membrane-associated target; delivery, stability, and field performance remain important constraints	[53]
Benzohydroxamic acid	Chitin deacetylases	*Verticillium dahliae*, *Fusarium graminearum*, *Fusarium oxysporum*, *Rhizoctonia solani*, *Puccinia striiformis* f. sp. *tritici*/plant infection assay	Biochemical; structural	In vitro assay; plant assay	Potted plants and Controlled plant systems	Anti-virulence	Anti-virulence activity may depend on host immune competence and disease pressure	[54]
VS#2-1	Chitin deacetylases	*Podosphaera xanthii*, *Botrytis cinerea*, *Penicillium digitatum*/plant infection assay	Biochemical	In vitro assay; plant assay	Potted plants and Controlled plant systems	Anti-virulence	Evidence appears less mature than structural CDA inhibitors	[55]
J075-4187	Chitin deacetylases	*Aspergillus nidulans*, *Fusarium graminearum*, *Aspergillus flavus*, *Botrytis cinerea*, *Fusarium oxysporum* f. sp. *cucumerinum* and *Saccharomyces cerevisiae*	Biochemical	In vitro assay	No whole-plant/field evidence	Anti-virulence	Further target-engagement and field-level validation are needed	[56]
FY21001	Pathogen effector-host immune protease interface: MoErs1–OsRD21	*Magnaporthe oryzae*/rice blast-related assay	Biochemical; structural	In vitro assay; plant assay; field trial	Detached tissues, potted plants and disease-control under field conditions	Anti-virulence	Strong proof of concept for effector-interface targeting; durability may be affected by effector variability	[57]
SOD9.14F and SOD9.26F	*B. cinerea* superoxide dismutase 1 BcSOD1	*Botrytis cinerea*/gray mold-related assay	Biochemical	In vitro assay; plant assay	Detached tissues	Host-directed/dual-action	Aptamer modality faces delivery, stability, and cost constraints for field use	[58]
C_18_-SMe_2_^+^	Mitochondrial function	*Zymoseptoria tritici*, *Magnaporthe oryzae*, *Ustilago maydis*/plant disease-related assay	Biochemical	In vitro assay; plant assay	Potted plants and Controlled plant systems	Host-directed/dual-action	Dual activity complicates selectivity assessment and deployment optimization	[59]

Validation categories: Biochemical = target-related enzyme, binding, activity, or target-engagement assays; Structural = experimentally resolved structures or experimentally supported structure-guided binding models; In vitro = fungal growth, germination, or pathogenicity-related assays outside the host plant; Plant assay = infection or disease-control assays using detached tissues, seedlings, potted plants, or controlled plant systems; Field trial = disease-control evaluation under field or field-like agricultural conditions. These categories describe reported evidence and do not imply equivalent developmental maturity.

**Table 3:** A practical evaluation framework for targeted fungicide candidates under agricultural conditions.

Evaluation Stage	Key Question	Recommended Assessment	Decision Relevance
Target or pathway relevance	Is the target linked to fungal growth, infection, virulence, or host defence modulation?	Genetic validation, infection-stage expression, loss-of-function or gain-of-function analysis, pathway-position analysis	Distinguishes biologically relevant targets from incidental or weakly connected targets
Biochemical target validation	Does the compound engage the proposed target or pathway?	Enzyme inhibition, binding assays, target-engagement assays, protein–compound interaction studies, structure-supported binding models	Confirms that activity is mechanistically linked to the proposed target
Cellular or in vitro activity	Does the compound affect fungal growth, development, infection-related morphogenesis, or virulence-associated processes?	Mycelial growth, spore germination, appressorium formation, penetration-related assays, stress-response assays, virulence-factor assays	Identifies early biological activity and separates fungitoxic from anti-pathogenicity effects
Selectivity and non-target profile	Is the effect selective for the pathogen, fungal group, or pathosystem?	Comparison with plant homologues, beneficial fungi, non-target microorganisms, and representative host tissues or cells	Evaluates taxonomic or pathosystem selectivity and potential non-target toxicity
Plant disease-control assay	Does the compound reduce disease in a plant system?	Detached tissue, seedling, potted-plant, greenhouse-like, or postharvest assays depending on the pathosystem	Links target engagement and in vitro activity to disease-control performance
Crop safety and physiological cost	Does the compound affect host growth, development, yield-related traits, or defence homeostasis?	Phytotoxicity assays, growth and biomass measurements, chlorosis or necrosis scoring, defence-marker monitoring, yield-related observations where applicable	Screens for host damage and unintended defence-associated costs
Formulation and delivery feasibility	Can the compound reach the relevant infection site at an effective dose?	Solubility, stability, uptake, surface retention, tissue penetration, compatibility with adjuvants, formulations, and tank-mix partners	Determines whether biological activity can be translated into practical application
Environmental robustness	Is the compound stable and effective under agriculturally relevant conditions?	UV stability, rainfastness, temperature stability, nuclease or protease degradation where relevant, persistence on plant surfaces, degradation profile	Evaluates whether efficacy is likely to persist outside controlled laboratory settings
Resistance risk	How likely is the target or pathway to lose sensitivity under selection?	Target conservation analysis, spontaneous mutant screening, cross-resistance testing, fitness-cost assessment, monitoring of target-site variation, virulence-factor redundancy, and compensatory infection phenotypes	Supports resistance-management planning and deployment durability
Compatibility with existing programmes	Can the candidate be integrated with current disease-management strategies?	Mixture or rotation compatibility, interaction with existing fungicides or biologicals, spectrum-of-control assessment	Determines practical fit within integrated disease-management programmes
Greenhouse and field validation	Does the compound provide reproducible disease control under realistic conditions?	Dose–response assays, greenhouse trials, multi-location or multi-season field trials where available, comparison with standards	Provides the strongest evidence for translational potential and deployment readiness

This framework is intended as a flexible checklist rather than a mandatory linear pipeline, and the relative importance of each stage may vary among fungal biology-targeted, anti-virulence, and host-directed or dual-action strategies.

## Data Availability

No new experimental data were generated in this study. The structural survey summarized in Figure 2 was based on publicly available entries from the Protein Data Bank (PDB), with retrieval details provided in the Figure 2 legend.
